# Evolutionary optimality in sex differences of longevity and athletic performances

**DOI:** 10.1038/srep05425

**Published:** 2014-06-24

**Authors:** Hiromi Asanuma, Satoshi Kakishima, Hiromu Ito, Kazuya Kobayashi, Eisuke Hasegawa, Takahiro Asami, Kenji Matsuura, Derek A. Roff, Jin Yoshimura

**Affiliations:** 1 Department of Mathematical and Systems Engineering, Shizuoka University, Hamamatsu, Shizuoka 432-8561, Japan; 2 Graduate School of Science and Technology, Shizuoka University, Hamamatsu, Shizuoka 432-8561, Japan; 3 Laboratory of Insect Ecology, Graduate School of Agriculture, Kyoto University, Kyoto 606-8502, Japan; 4 Department of Ecology and Systematics, Graduate School of Agriculture, Hokkaido University, Sapporo, Hokkaido 060-8589, Japan; 5 Department of Biology, Shinshu University, Matsumoto, Nagano 390-8621, Japan; 6 Department of Biology, University of California, Riverside, CA 92521, USA; 7 Marine Biosystems Research Center, Chiba University, Kamogawa, Chiba 299-5502, Japan; 8 Department of Environmental and Forest Biology, State University of New York College of Environmental Science and Forestry, Syracuse, NY 13210, USA; 9 These authors contributed equally to this work.

## Abstract

Many sexual differences are known in human and animals. It is well known that females are superior in longevity, while males in athletic performances. Even though some sexual differences are attributed to the evolutionary tradeoff between survival and reproduction, the aforementioned sex differences are difficult to explain by this tradeoff. Here we show that the evolutionary tradeoff occurs among three components: (1) viability, (2) competitive ability and (3) reproductive effort. The sexual differences in longevity and athletic performances are attributed to the tradeoff between viability (survival) and competitive ability that belongs to the physical makeup of an individual, but not related to the tradeoff between survival and reproduction. This provides a new perspective on sex differences in human and animals: females are superior in longevity and disease recovery, while males are superior in athletic performance.

There are many sex differences in humans and animals^1^. The most well-known biological sex difference is sex-specific survivorship and longevity (life span or life expectancy at birth), where females live longer than males on average^2^,^3^,^4^. Another well-known gender difference is athletic performance in many sports, where males are usually superior in most comparable games^5^. These sex differences are suspected to have originated from two mechanisms: different functions associated with the sexual organs and optimality differences between sexes^1^. Examples of the former are various sex-specific traits such as menstrual periods, pregnancy and breast-feeding in females and ejaculation in males. The latter comprise various evolutionary (optimality) tradeoffs between individual survival and reproduction. The cost of reproduction in iteroparous (multiple bouts of reproduction) animals is a typical example of such tradeoffs^6^,^7^. However, some sex differences in humans are still difficult to explain by either the tradeoff between survival and reproduction, or by the sex-specific traits. We still have no clear scientific basis for these sex differences.

Here we discuss a tradeoff between viability (survival) and competitive ability in the physical makeup of an individual. Because competitive ability is an important factor for mate acquisition for males, competitive ability in the form of male-male combat is also considered a cost of reproduction^8^,^9^,^10^. In these cases, we often find sexual dichotomy in body size or the size of a body part. However, this tradeoff is distinctively different from the tradeoff between survival and reproduction, and is better explained under sexual selection (either mate choice or mating competition). Because males also allocate their efforts to reproduction, such as male sexual organs and male parental/family care, males also allocate the reproductive investment, as females.

The sex-specific differences in survival and longevity, and athletic performance can be explained by the difference in a tradeoff between viability and competitive ability. Because of the costs of giving birth (labor) and/or parental care, female reproductive success is more sensitive to survivorship than males, while male reproductive fitness is more determined by mate competition^11^,^12^,^13^,^14^. Because mate competition is usually contest competition, a high athletic ability is advantageous for mate competition in males.

To explain these sex-specific differences we first build an allocation model of individual effort (energy) among three major components: (1) viability, (2) competitive ability, and (3) reproductive effort. Next, we consider the tradeoff between viability and competitive ability, the first two components of which are related to the physical makeup of one's body, and evaluate the optimality (fitness) difference between the sexes. Following, we introduce various examples of sex differences in humans and animals. Finally, we discuss the generality of this tradeoff model, its applicability to other animals and its implications for gender differences in human society.

## Results

**Figure 1:**
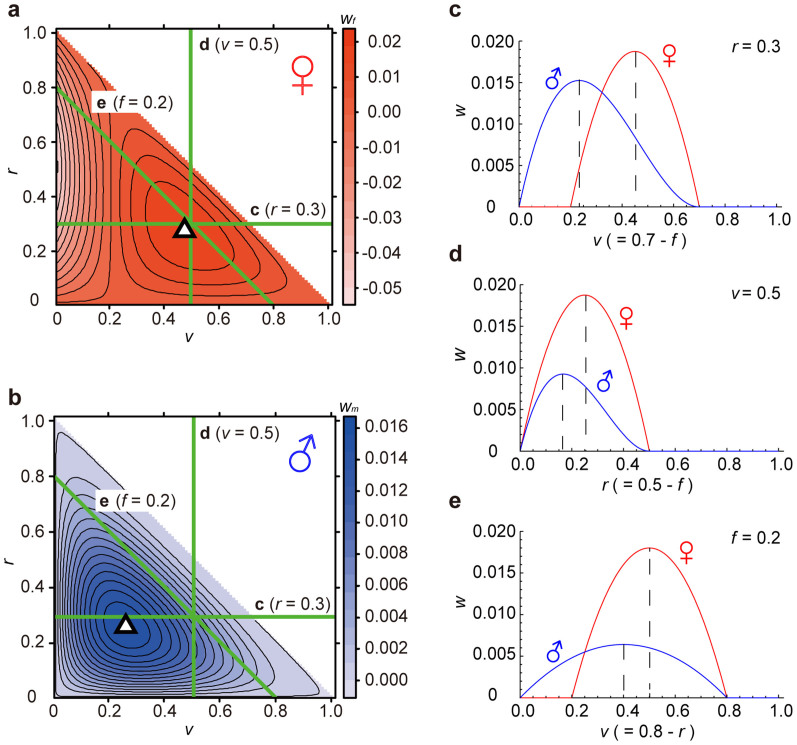
Model of a sex-specific fitness tradeoff between viability, competitive ability and reproduction. Assume the tradeoff among three components: viability *v*, competitive ability *f* and reproduction *r*, such that *v* + *f* + *r* = 1. We set *c* = 0.2 and *n* = 2, unless otherwise noted. (a,b) Fitness landscapes against *v*, *f*, and *r*. Because *f* = 1 – (*v* + *r*), *f* is automatically determined by the *v*-*r* plane, where 0 ≤ (*v*+r) ≤ 1 that corresponds 1 ≥ *f ≥ 0*. The open triangles indicates the optimal points (peaks). Three green lines in (a, b) corresponds (c–e). (a) Female fitness landscape, *w_f_* = (*v*-*c*)*fr* = (*v*-0.2)*fr*. (b) Male fitness landscape, *w_m_* = *v f^ n^r* = *v f*^2^*r*. (c) The tradeoff between *v* and *f*, assuming *r* = 0.3. (d) The tradeoff between *r* and *f*, assuming *v* = 0.5. (e) The tradeoff between *v* and *r*, assuming *f* = 0.2.

The sex difference in the fitness tradeoff among *v*, *f* and *r* results in the different optimal allocations between males and females (Fig. 1a,b). We consider the basic tradeoff of individual effort (energy) in early juvenile stages where reproductive effort is assumed to be negligible. For example, in juvenile humans before the age of 10 years, reproductive effort is almost limited to small-undeveloped sexual organs. Therefore, we assume the reproductive investment *r* is constant and small (Fig. 1a,b). Equations (2) and (3) inevitably lead to a sex difference in the optimal allocation of viability *v* and competitive ability *f*, irrespective of parameter values (Fig. 1c). In contrast, in late juvenile or adult stages, allocation to reproduction *r* becomes critical (Fig. 1d,e). Ignoring viability (survival) *v*, we get the tradeoff between competitive ability and reproduction (Fig. 1d)^11^. We also get the well-known tradeoff between survival and reproduction that incurs a cost of reproduction, assuming the effort for competitive ability *f* is constant (Fig. 1e).

## Discussion

**Figure 2:**
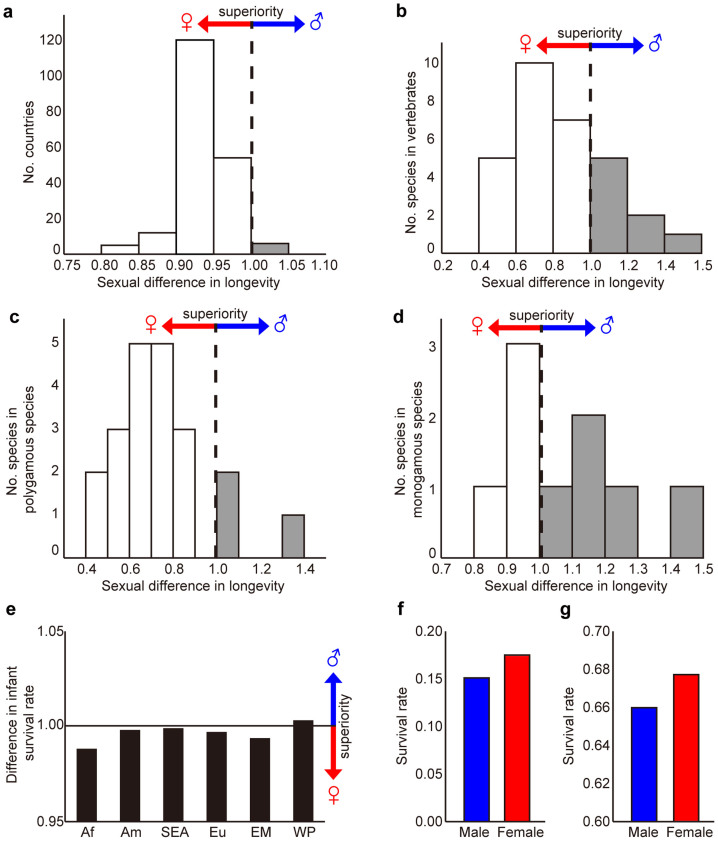
Examples of female superiority in humans and animals. (a–d) Sexual difference of superiority in longevity. The sexual difference in longevity is measured by the value of (male longevity)/(female longevity). (a) The number of countries plotted against sexual difference in human longevity^2^. (b) The number of species against sexual difference in longevity in 30 vertebrate species^15^. (c) The number of species against sexual difference in longevity in 21 polygamous species^15^. (d) The number of species against sexual difference in longevity in 9 monogamous species^15^. (e) Sexual differences in human infant survival rate in six regions^3^. The graphs show the values of (male infant survival)/(female infant survival). The survival rate of infant to reach age 1 is used for each region. Regions are Africa (Af), Americas (Am), South-East Asia (SEA), Europe (Eu), East Mediterranean (EM), and Western Pacific (WP). (f) Five-year survival rate of people suffering cancer after operation in Japan^17^. Female survival rate is significantly larger than male survival rate (chi-square test: *p* = 0.0031). (g) 28-day survival rate of people suffering myocardial infarction after operation in Sweden^18^. Female survival rate is significantly larger than male survival rate (chi-square test: *p* = 8.7 × 10^−6^). (a–d) The histogram of (male longevity)/(female longevity) is shown. (a–e) Male is superior if the difference > 1.00, while female is superior if the difference <1.00.

A tradeoff between viability *v* and competitive ability *f* is evident in empirical data of humans and some vertebrates. First, human females exhibit greater longevity in almost all countries (Fig. 2a)^2^. Out of the total 30 species of vertebrates, 22 species exhibit greater longevity in females (Fig. 2b)^15^. Female superiority becomes more evident (18 species out of 21 species; 86%) in polygamous species where there is severe competition among males for females (Fig. 2c)^15^. In contrast, in monogamous species where male competition is less severe, the number of species with longer male longevity (4 species out of 9 species; 44%) becomes slightly more than a half (Fig. 2d)^15^. If we limit the data to primates, 5 species out of 6 exhibit longer female longevity^16^.

Juvenile survivorship also exhibits similar trends. In human infants survival rates to age 1 are lower in males in all the regions of the world except the Western Pacific Region, irrespective of the regional differences in actual mortality (Fig. 2e)^3^. Female superiority is also seen in disease recovery in two serious lethal diseases, cancer and myocardial infarction. In these cases, survival rate after an operation is much higher in females, e.g., 5-year survival in cancers (Fig. 2f)^17^ and 28-day survival in myocardial infarction (Fig. 2g)^18^.

**Figure 3:**
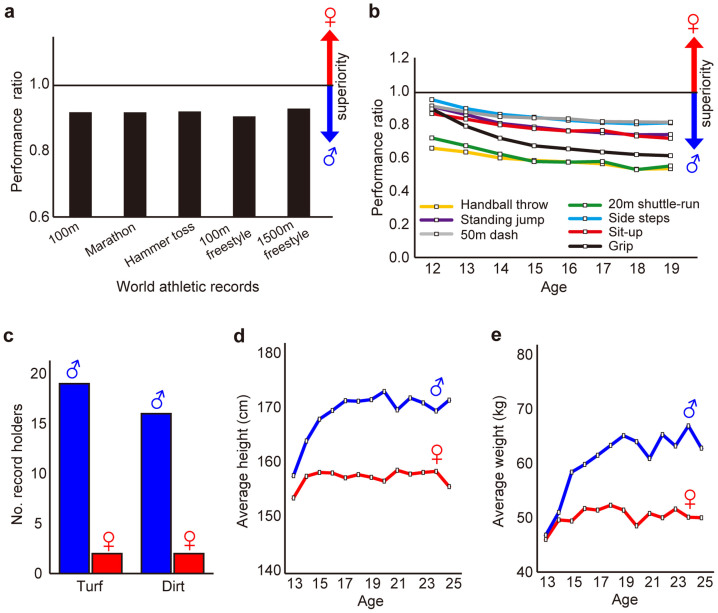
Examples of male superiority in competitive ability. (a) The performance ratio between sexes in several world athletic records^5^,^19^. The ratio of (male record)/(female record) is shown for 100 m running, marathon, 100 m freestyle swimming and 1500 m freestyle swimming. The ratio of (female record)/(male record) is used for hammer toss, because sexual superiority is reversed in this game. (b) Temporal changes in the performance ratios between sexes in the average juvenile (aged 12–19) in Japan^20^. The ratio of (female record)/(male record) is used for all games except the 50 m dash for which the ratio of (male record)/(female record) is used to align the measure of sexual superiority. (c) The numbers of record holders for each sex in horse races^21^. The turf and dirt courses are shown separately. (d) Male and female average heights in Japanese human populations between 13–25 years old^20^. (e) Male and female average weights in Japanese human populations between 13–25 years old^20^. (a,b) Female is superior if the performance ratio > 1.00, while male is superior if the performance ratio <1.00.

In contrast to viability (survival), male superiority is expressed in many athletic records and physical bodily makeup. The world records exhibit male superiority in at least three athletic games and two swimming races that are comparable between the sexes (Fig. 3a)^17^,^19^. The same trends are seen in the average athletic records of young (age 10–19) in Japan (Fig. 3b)^20^. Male superiority is also found in horse races in Japan, where most track record holders are males in both turf and dirt courses (Fig. 3c)^21^. These records indicate male superiority in instantaneous forces and physical (muscle) strengths. Male superiority is also recognized in the physical bodily makeup in humans. In the 13- to 25-year age classes in Japan, the average height is always higher in males (Fig. 3d) and the average weight is always heavier in males (Fig. 3e)^20^. The athletic events themselves probably originated, in part, as a display by males to females.

The current model shows the tradeoff among three components: viability *v*, competitive ability *f*, reproductive effort *r*. Therefore we can consider three tradeoffs: (1) *v* vs. *f*, (2) *v* vs. *r*, and (3) *f* vs. *r*. Traditionally, many sexual differences have been considered to originate from the tradeoff between survival and reproduction^22^. This means the second tradeoff between *v* vs. *r*. For example, the cost of reproduction means the tradeoff between current reproduction and future survival (viability). Because current survival increases future reproduction, it is essentially the tradeoff between current reproduction and future reproduction^23^. Trivers also pointed out that some sex differences might have evolved from the tradeoff between competitive ability (male-male competition) and reproduction (as a form of parental investment)^11^. This means the third tradeoff between *f* vs. *r*. Note here that male strategies can be variable between *f* vs. *r*: those mating with many girls (“playboys”) and those caring for their family (devoted husbands).

Both reproduction and competitive ability can be considered a cost of reproduction, because these factors can decrease the survival (viability) of an individual^11^. This means the tradeoff between *v* vs. (*f*, *r*), indicating the combination of the second and third tradeoffs. However, the tradeoff between viability (survival) and competitive ability had not been considered explicitly. This is probably because competitive ability was usually categorized as a part of the cost of reproduction that reduces parental survival. Therefore, we could not consider the first tradeoff between *v* vs. *f*, assuming *r* is constant. The uniqueness of this tradeoff is that it does not involve the reproductive effort of an individual. Here both *v* and *f* constitute the bodily makeup of an individual apart from reproduction. If a body is made durable and long-lasting, viability *v* increases. In contrast, if it is made responsive and powerful (in strength), competitive ability *f* increases.

We should note that before sexual development, reproductive investments should be very small. During an early stage of childhood (less than 10 year olds), there is almost no reproductive allocation of total energy, except for very small sexual organs that are about the same size between sexes. The tradeoff with reproductive effort is not so important at this stage, even though there are clear but slight differences in ontogeny, e.g., females mature earlier. In the current model we focus on the tradeoff between survival (viability) and competitive ability. Both of them are important even during juvenile stages as suggested by differences in infant survival (until age 1) (Fig. 2e). This implies that the sexual differences at juvenile stages should not be related to the allocation for reproduction.

In the current model the above tradeoffs are included in the tradeoff among the three components: survival, competitive ability of an individual and reproduction. As Trivers suggested^11^, male-male competition is expected to be stronger in animals with polygamy than with monogamy. Polygamous animals may sacrifice either parental investment or viability, or both to increase competitive ability against other males. We, therefore, expect female longevity to be greater in polygamous species compared with monogamous species (Fig. 2c,d).

The present sex difference should be fundamental, since it originates from the difference between the costs of pregnancy and birth in females and mating success in males (Equations 2 and 3). Therefore, the logic is equally applicable to any promiscuous species e.g., *Drosophila* and many other solitary insects^11^,^12^,^24^,^25^. Some dichotomies of body size in hominids and primates may also be explained by this tradeoff^26^,^27^.

The tradeoff described above implies that males are more competitive and physiologically fragile (greater mortality) and females are cooperative and physiologically tough (tolerant). In this sense, females may be considered far superior to males in the coevolving world of organisms, unlike the traditional discriminatory view of gender differences against females.

## Methods

### Ethics Statement

The data involving human participants and animals are gathered solely from the public domain (web sources) as indicated in the references cited.

### Tradeoff Model

We assume the basic biological tradeoff of individual effort (energy) among viability *v*, competitive ability *f* and reproductive investment *r* is an additive function such that 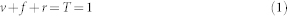
where the total effort, *T,* is assumed constant (Fig. 1). Note that competitive ability includes both instantaneous forces and muscle strengths that are expressed in athletic performance, and that reproductive investment includes parental care.

Fitness is assumed to be a compound function of survival and reproductive success^28^. The latter, reproduction success, consists of reproductive output (the number of offspring), with which male mating success usually correlates. Survival is a function of viability, *v*, mating success, (i.e. competitive ability *f*), and reproductive output, (i.e. reproductive investment *r*). Because the fitness consequences of *v*, *f* and *r* are different between sexes, we define the fitness of males and females separately. For females, which have to survive labor and parental care, the female fitness *w_f_* is defined as 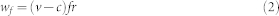
where *c* is the cost necessary to survive until the end of the prenatal (time to and including birth) period and parental care. This optimization model can easily be solved analytically using Lagrange multipliers. The optimal point is (an open triangle in Fig. 1a) 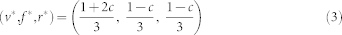
In contrast, since males have to fight with other males to acquire mates (females), competitive ability becomes more important in males than females. Therefore, we define male fitness, *w_m_*, as 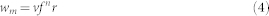
where the power *n* (>1) represents the severity of male-male competition. Note that the contribution of *f* to male fitness, *w_m_*, becomes larger when male competition is severe (*n* ≫1). The reason for the power in equation (4) is that the chance of mating should increase abruptly with *f*. Here, the survival cost *c* only is assumed only in female fitness, because the female reproductive success is only guaranteed after her child becomes independent, while, male reproductive success becomes at least possible right after child birth. The optimum for male (equation (4)) can be also solved analytically in the same manner of the female fitness (equation (2)). The peak is (an open triangle in Fig. 1b) 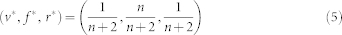


## Author Contributions

H.A., S.K., H.I. and J.Y. conceived the study. S.K. and H.A. collected data. H.A., H.I., K.K., D.R. and J.Y. built the mathematical model. E.H., T.A. and K.M. contributed the interpretation of the model. H.A., S.K. and J.Y. wrote the manuscript.
